# Defective data: statistics, disability, and eugenic sterilisation in interwar Britain

**DOI:** 10.1017/mdh.2025.10014

**Published:** 2025-07-18

**Authors:** Alex Aylward, Coreen McGuire

**Affiliations:** 1 Faculty of History, University of Oxford, Oxford, UK; 2 Department of History, Durham University, Durham, UK

**Keywords:** Eugenics, Sterilisation, Mental Deficiency, Disability, Data, Propaganda

## Abstract

This article is concerned with the history of eugenic sterilisation in Britain through the 1920s and 1930s. In this period, the Eugenics Society mounted an active but ultimately unsuccessful campaign to legalise the voluntary surgical sterilisation of various categories of people, including those deemed ‘mentally deficient’ or ‘defective’. We take as our explicit focus the propaganda produced and disseminated by the Eugenics Society as part of this campaign, and especially the various kinds of data mobilised therein. The parliamentary defeat of the Society’s Sterilisation Bill in July 1931 marks, we argue, a significant shift in the tactics of the campaign. Before this, the Eugenics Society framed sterilisation as a promising method for eradicating, or at least significantly reducing the incidence of, inherited ‘mental defect’. Subsequently, they came to emphasise the inequality of access to sterilisation between rich and poor, (re)positioning theirs as an egalitarian campaign aimed at extending a form of reproductive agency to the disadvantaged. These distinct phases of the campaign were each supported by different kinds of propaganda material, which in turn centred on very different types of data. As the campaign evolved, the numbers and quantitative rhetoric which typified earlier propaganda materials gave way to a more qualitative approach, which notably included the selective incorporation of the voices of people living with hereditary ‘defects’. In addition to exposing a rupture in the Eugenics Society’s propagandistic data practices, this episode underscores the need to further incorporate disabled dialogues and perspectives into our histories of eugenics.

Eugenics seeks for quantitative results. It is not concerned with such vague words as ‘much’ or ‘little’, but endeavours to determine ‘how much’ or ‘how little’ in precise and trustworthy figures.

–Francis Galton^1^

## Introduction

Eugenics is the project of improving the biological ‘quality’ of a population by controlling or influencing who does, and who does not, reproduce. The movement, so named, traces its beginnings to the nineteenth-century research and writings of English polymath Francis Galton.^2^ Though its origins are British, the eugenic project would be more fully and horrifically realised elsewhere, most infamously in Nazi Germany.^3^ When measured against the ambitions of its supporters and compared with its overseas equivalents, the eugenics movement in Britain appears as something of a failure. In particular, the Eugenics Society – the principal body agitating for explicitly eugenic legislation in Britain through the twentieth century – was largely unable to exert meaningful influence on government policy.^4^ Notably, and in contrast to developments in the United States, Scandinavia and elsewhere, no legislation permitting eugenic sterilisation was ever passed in Britain. This was not for want of trying. Through the 1920s and 1930s, the Eugenics Society poured significant energies and resources into a vigorous campaign to legalise voluntary sterilisation on eugenic grounds, for individuals exhibiting a range of purportedly hereditary ‘defects’.

In this article, we examine some central yet overlooked aspects of that campaign. By doing so, we draw attention to the different epistemic and effective qualities of data and show how their differing presentation as quantitative or narrative variously worked to construct disease as disability. In the first half, we highlight the extent to which the early part of the Eugenics Society’s campaign was dominated by numbers, and one number in particular – the prediction, associated with eugenicist and noted statistician Ronald Fisher, that sterilisation of individuals deemed ‘mentally defective’ would result in a 17.4 per cent reduction in the affliction’s incidence within just one generation. This curiously precise prediction at first energised the leadership of the Eugenics Society, but soon came to be viewed as a weak point in their arguments as critics within and beyond the medical profession attacked its apparently unrealistic and unfounded assumptions. Before long, the once-ubiquitous number was expunged from all Society propaganda. This case study, of the rise and fall of a number, highlights both the strategic attractions and practical shortcomings of what we call ‘quantitative rhetoric’. On the one hand, Fisher’s number brought a welcome exactness to the Society’s case for sterilisation. On the other hand, it exposed them to the challenge that their arguments were detached from the realities of the lives and care of ‘defective’ people.

If the first half of this essay foregrounds numbers, the second centres the people whose sterilisation was deemed desirable. Just as disabled people’s rich and complex lives were obscured by impersonal numerical predictions such as Fisher’s, so too has their agency been largely absent from histories of sterilisation campaigns.^5^ Across the 1930s, dozens of people – disabled and non-disabled – wrote letters to the Eugenics Society seeking advice and help in securing sterilisation procedures for themselves or their loved ones. In some cases, they got them, aided logistically and financially by the Eugenics Society. A failure to secure the passage of eugenic sterilisation legislation does not, it turns out, entail a complete absence of ‘eugenic’ sterilisations.

In addition to revealing the Eugenics Society’s activities in arranging private sterilisation procedures through the 1930s, we suggest that this correspondence underlines a need to take disabled perspectives more seriously in interpreting the Eugenic Society’s shifting approach to promoting new sterilisation legislation, and in telling the history of eugenics more generally. Though peripheral in most histories of the sterilisation campaign in interwar Britain, disabled individuals were important actors. Not just intended targets of sterilisation, disabled people were also consumers of, and in some cases contributors to, pro-sterilisation eugenic propaganda; their lives and their letters provided a new data source within the Eugenics Society’s renewed campaign. Our hope is that, by examining some of the ways disabled people engaged with eugenic ideas and organisations in interwar Britain, the present essay can contribute to an ongoing ‘disabling’ of eugenics scholarship.

Taken together, these two case studies – of numbers and letters, respectively – reveal several significant transformations in the Eugenics Society’s campaign tactics from the early 1930s. Firstly, there occurred a notable expansion of scope. The campaign up to and including the introduction of a Sterilization Bill before parliament in 1931 had focused almost exclusively on the problem of ‘mental defect’. The Bill’s resounding defeat prompted not a retreat but a reorientation, as the Eugenics Society multiplied its target to include a far broader range of physical as well as intellectual disabilities. Attendant to this was a shift in the principal reasons they offered for supporting the passage of sterilisation legislation, from initially emphasising its necessity in combatting a growing epidemic of ‘mental deficiency’, to an ‘equality’ argument wherein legislation was required not primarily to deal with disease or defect, but instead to democratise access to a medical procedure presently available only to the relatively privileged.

Finally, and relatedly, we see an abrupt break in the kinds of data given prominence within the Eugenics Society’s propaganda output, from the quantitative to the qualitative. Numbers, deemed guilty of abstracting away from the particularities of the people they enumerated, gave way to narratives. Disabled people and their stories – their accounts of the legal and financial barriers they faced in accessing the sterilisation procedures they and their families desired – became key qualitative data points, collected and mobilised within a renewed campaign to legalise the curtailment of their fertility.

### Eugenics by numbers

We routinely use numbers to measure, define, and categorise people. In the medical and human sciences, numbers of various kinds are deployed with the intention of capturing real and relevant differences among people, and thus helping to guide our understanding or treatment accordingly. Numbers represent, but they also remake. Mobilised to delineate the ‘normal’ from the ‘pathological’, the ‘able’ from the ‘disabled’ and, even more problematically, the ‘deserving’ from the ‘undeserving’, numbers *make* a difference.^6^

Numbers are powerful, then, and can be harmful. So much is plain in the case of eugenics, which lent a scientific veneer to discrimination and caused untold suffering and violation of bodies and rights. It did so, often, through the invocation of numbers. The rise of eugenics was concomitant with the emergence of modern statistical methods, as the likes of Francis Galton, Karl Pearson, and Ronald Aylmer Fisher developed new tools for analysing biological variation, measuring correlation between parents and offspring, and parsing the respective roles of nature and nurture.^7^ When attention turned to the practical problem of assessing the ‘racial’ value of the population, quantification – through the collection and analysis of anthropometric and demographic data – remained central.^8^ Eugenic policy proposals, meanwhile, often targeted statistically defined portions of the populace, not least the notorious ‘submerged tenth’, whose supposed combination of overzealous breeding and inferior genetics was posited as a major existential threat.^9^ Eugenics – academic and practical – has always been a numbers game.

An often-overlooked function of numbers, within the eugenics historiography if not elsewhere, is to *persuade*. During its interwar sterilisation campaign, Britain’s national Eugenics Society made prominent and tactical use of designated data and selected statistics, with the explicit intention of winning over, or overwhelming, opponents. Previous scholarship on Britain’s eugenic sterilisation movement engages with this phenomenon only indirectly. Historians have examined various aspects of the campaign, including the vacillation of genetics experts on the causes and inheritance of ‘mental deficiency’,^10^ the underlying ideologies which guided the variety of medical, social and political ‘solutions’ proposed by reformers,^11^ and more recently, the Eugenics Society’s ineffectual parliamentary politicking.^12^ These scholars routinely quote (and as we shall see, occasionally misquote) the headline figures which were pushed by their historical actors – sterilisation advocates and detractors alike. They do so, usually, with little reflection on where these numbers came from, their functions and their effects, and on why the people involved attached such significance to them.

The deployment of numbers to persuade is, of course, commonplace. Governments routinely gather, solicit, and endorse numbers in their attempts to influence citizen behaviour and justify their policies.^13^ In our personal and professional lives, we often seek the support of numbers when advocating for one among a range of possibilities. Perhaps we do not remark upon the ‘numerical rhetoric’ of eugenics because it seems so obvious.^14^ The next section, though, urges a more conscious examination of eugenicists’ number talk, through a case study of a single number which assumed, albeit briefly, a particularly significant role within the Eugenics Society’s sterilisation campaign. What is notable here is not simply that eugenicists reached for numbers in making their case for sterilisation, but more so how suddenly and decisively they *stopped* doing so. The rapid rise and even more rapid fall of their chosen number underlines the potential for numerical rhetoric to be impotent, as well as persuasively powerful.^15^ This case, then, helps us to think through some of the reasons why we sometimes place trust, and at other times *mis*trust, in numbers.^16^

### The rise and fall of a number

*The Lancet*’s issue for 21^st^ June 1930 included a Report to the Lancashire Asylums Board on the problem of the ‘Sterilisation of the Unfit’, authored by Dr Frank A. Gill, medical superintendent of the Calderstones Certified Institution for Mental Defectives in Whalley, Lancashire. While Gill believed surgical sterilisation a safe procedure, desirable and therapeutically justifiable in a range of circumstances, he had major reservations concerning what he saw as the ‘primary and chief claim’ of sterilisation advocates, namely that it will ‘in time, materially reduce the numbers of the insane and mental defectives, and so ultimately relieve the community of the burden of their maintenance’. Sterilising people certified as ‘defectives’ would do little to affect their numbers, Gill reasoned, given that most of these people were themselves the children of mentally ‘normal’ parents. Personal experience of asylum work convinced Gill on this point. Among the 2,500 ‘inmates’ of one Lancashire institution, he explained, ‘[v]ery few parents are certifiable defectives, the great majority are decent self-supporting members of the community, whom no sterilisation laws could touch or seek to touch’.^17^

At just this time, the Eugenics Society was mobilising a campaign to legalise the sterilisation of ‘mental defectives’ on the grounds of racial improvement. Founded in 1907, the Society had in its early years lobbied successfully for the passage of the Mental Deficiency Act in 1913.^18^ While this act provided for the institutionalisation in ‘colonies’ of individuals deemed ‘feeble-minded’, many within the eugenics movement thought it time to go further. Inspiration came from the United States, where the sterilisation laws trialled by various states had recently gained constitutional legitimacy via the widely publicised *Buck v. Bell* ruling of 1927.^19^ Back in the United Kingdom, the 1929 ‘Wood’ Report of the Interdepartmental Committee on Mental Deficiency sounded the alarm on a veritable epidemic of mental deficiency (the number of Britain’s certified ‘defectives’ having apparently almost doubled over two decades), and urged that sterilisation be seriously considered as a cure for this growing social ill.^20^

Buoyed by these developments, the Eugenics Society hurriedly drafted a bill advocating the provision of voluntary sterilisation of ‘defectives’ on eugenic grounds, and set about drumming up the public and political support necessary to see it into and eventually through parliament. One of the major obstacles to the success of their campaign, as they saw it, was a widespread opposition to sterilisation among medical men, epitomised in Dr Gill’s hostile report. Indeed, *The Lancet* and other mainstream medical journals had long proven to be a thorn in the movement’s side.^21^ *The British Medical Journal*, in particular, was known for publishing ‘sarcastic and ill-informed comments about eugenics’ and the Eugenics Society’s President, the former civil servant Sir Bernard Mallet, thought it high time the venue was ‘pulled up a bit’.^22^ To this end, senior members of his Eugenics Society penned letters to both journals, seeking to correct various mischaracterisations of the eugenic case for sterilisation apparently rife in the medical press, and to combat Dr Gill’s emphatic claim that ‘if every certifiable mental defective had been sterilised 20 or 30 years ago it would have made little appreciable difference to the number of defectives existing to-day’.^23^

In making their case, the letters leant heavily on numbers, or rather one number in particular. ‘One of us has shown,’ read the submission to *The Lancet*, published July 19 1930, ‘that if all the defectives in the community could be prevented from having children the effect would be, even on the most unfavourable genetic and social assumptions with regard to defectiveness, to reduce the incidence of mental defect by as much as seventeen per cent. in one generation’.^24^ The ‘one of us’ was Ronald Aylmer Fisher, a statistician of growing repute, recently made a Fellow of the Royal Society for his contributions to mathematical statistics. The seventeen per cent figure came from an article which Fisher had published six years earlier, itself a critical response to a set of projections made in 1917 by the Cambridge geneticist Reginald Punnett. Punnett, as did almost all geneticists of the time, assumed that mental deficiency was heritable and caused by a Mendelian recessive.^25^ Only those individuals who inherited a double dose of the faulty factor – one from each parent – would exhibit the trait, while those with just one copy would escape the affliction but could pass on their hereditary taint to future generations. This propensity of the defect-making factor to lie hidden in otherwise ‘normal’ stock posed a grave problem for its eradication through eugenic selection. Punnett calculated that a programme of sterilisation or segregation aimed at eradicating mental defect was doomed to failure. To reduce the incidence tenfold, from one in one hundred to one in 1,000, it would require twenty-two generations of sterilising all manifest ‘defectives’. Further reduction from one per 1,000 to one per 10,000 would require sixty-eight generations. From one in 10,000 to one in 1000,000: 216 generations. No real progress in eliminating the affliction could be expected, Punnett concluded, for ‘about 8000 years’.^26^

Punnett was a card-carrying eugenicist. But his projections, unsurprisingly, were seized upon by the movement’s critics, mobilised in anti-sterilisation pamphlets produced by the Central Association for Mental Welfare, and by detractors in a well-publicised debate on sterilisation at a 1923 meeting of the British Medical Association.^27^ Encouraged by Eugenics Society leadership, Fisher attempted to stem the flow of ‘anti-eugenic propaganda’ via a brief article in the July 1924 number of *The Eugenics Review*. In ‘The Elimination of Mental Defect,’ Fisher did two things. On the one hand, he took issue with Punnett’s starting assumptions, which he pointed out were peculiarly unfriendly to the promise of eugenic intervention. Punnett, for instance, had assumed random mating, whereas in fact ‘defectives’ were far more likely to reproduce with other ‘defectives’ than were ‘normal’ members of the population. This *non*-random pattern of mating would render sterilisation far more effective than Punnett calculated. At the same time, Fisher pointed out that a simple re-presentation of the results showed that, from the eugenicist’s point of view, Punnett’s numbers were not so gloomy after all. Rather than tabulating the number of generations necessary to effect consecutive tenfold decreases, Fisher set, on Punnett’s assumptions, the percentage decrease one could expect after each generation. After three generations, the overall reduction would be more than forty per cent, and in just one generation, Fisher explained, ‘the load of public expenditure and personal misery caused by feeblemindedness, if this is the particular defect considered, would be reduced by over 17 per cent.’ – 17.4, to be exact.^28^

Over the next few years, the Eugenics Society reprinted and distributed ‘thousands’ of copies of Fisher’s article, which it came to regard as ‘one of our most useful pamphlets’.^29^ As the campaign to legalise eugenic sterilisation gathered pace, the Society’s President Bernard Mallet called upon Fisher to produce for them a pamphlet setting out the argument ‘at greater length than in the short paper we have at our disposal’. ‘I should rather like,’ he explained,

something technical and rather difficult to understand which would show to medical men that genetical problems are rather more complicated than they glibly assume! A mathematical formula or two would be useful in this direction. Do you think you could perhaps do this? The medical subcommittee were unanimous that it would be of the utmost value to us.^30^

Though Fisher did not produce a further pamphlet along these lines, his headline statistic would in any case recur across the propaganda materials produced by the Eugenics Society’s newly established Committee for Legalizing Eugenic Sterilization, cited repeatedly and reverentially. The Committee produced a major pamphlet setting out its policy proposals and attempting to head off potential objections, with thousands of copies printed and distributed across the summer of 1930. In it, Fisher’s argument and his seventeen per cent estimate figured centrally.^31^ Numbers were weaponised, unabashedly. Coming from one of the world’s leading statisticians, they held particular sway. Copiously and consciously deployed, Mallet hoped that Fisher’s numbers would be the key to the campaign’s success. He was wrong.

At the height of the propaganda blitz, in July 1930, Mallet professed to Fisher that his numeric estimate was ‘one of the chief weapons at our disposal’.^32^ Things soon changed, however, and by October Fisher would observe:

whereas two months ago every member of the committee would have thought it useful to quote the result as a scientifically based opinion on a difficult subject, the validity of which has not been contested, at the present moment most members or all would be afraid to mention it.^33^

As 1930 drew to a close, and stocks of their pamphlet dwindled, the Committee for Legalising Eugenic Sterilisation moved to issue a second edition. This time, seventeen per cent was nowhere to be seen. What happened?

In the weeks and months following its initial publication, the Committee’s pamphlet had stoked significant interest, and not a little controversy. Much of the disapproval focused on Fisher’s figure. On August 16^th^, *The Lancet* published an even-handed summary of the pamphlet’s contents, but an accompanying editorial observed the following:

Although R. A. Fisher calculates that, if all primary aments were prevented forthwith from breeding, the incidence of mental deficiency would be reduced by 17 per cent. in a single generation, other authorities think that in practice sterilisation would not have much appreciable effect for a far longer period.^34^

One such authority was Alfred Frank Tredgold, psychiatrist and Chairman of the Central Association for Mental Welfare’s (CAMW) Medical Committee. Years of interacting with ‘defectives’ and their families had convinced Tredgold that of those individuals certified as ‘mentally deficient’, only around one in twenty, or five per cent, were born to parents similarly certified. Hence, he argued, any scheme of sterilising certified ‘defectives’ could only hope to affect a five per cent reduction in a generation, and given that the Eugenics Society advocated *voluntary* (rather than compulsory) sterilisation, the figure in practice would be substantially lower. While Mallet had banked on his authority *qua* statistician, Fisher’s lack of practical experience working with institutionalised people ensured that, among asylum practitioners, his figure lacked credibility. In fact, according to Tredgold, it was an ‘absurdity’.^35^

Carlos Paton Blacker, newly appointed as the Society’s General Secretary, was the man tasked with meeting recalcitrant medical bodies and thrashing out common ground on sterilisation. Fisher’s number, and the pushback it provoked from Tredgold and others, caused him no end of problems. What is more, its originator offered little by way of useful assistance. As Blacker put it to the biologist and writer Julian Huxley, another Eugenics Society bigwig busily banging the sterilisation drum in the national press:

I have had a certain number of difficulties with Dr. Fisher over this subject. It now transpires that he only intended his estimate of seventeen per cent to be an academic calculation to correct the misleading construction placed upon Punnett’s original article which has been quoted ad nauseum by opponents of sterilization. I have not yet been able to find out what figure he is prepared to defend as being possible to achieve in practice. For our purposes academic calculations based on Punnett’s admittedly erroneous premises have not much interest.^36^

He urged, therefore, that Huxley make ‘as little reference as possible’ to the prediction in any future articles, and pressed successfully for the figure’s exclusion from the next edition of the Committee’s pamphlet. When Mallett, initially so enthused with the calculation and its argumentative potential, broke news of the decision to Fisher, he emphasised that the numerical prediction signified ‘the main thing that divides us,’ and that were they to drop it, ‘it seems quite likely that the CAMW might endorse our arguments and support our proposals’.^37^

Social numbers teem with ambiguities, and this is true even (perhaps especially) of remarkably precise numbers such as 17.4 per cent. The ambiguity of Fisher’s number – was it an ‘academic’ calculation or a concrete prediction? – rendered it flexible, allowing the Eugenics Society to mobilise it towards ends other than those for which it was initially intended. The same ambiguity, though, left it open to misinterpretation and attack. We might think it ironic that the CAMW railed so vehemently against the 17.4 per cent figure, given how enthusiastically they had previously pushed Punnett’s projections, of which Fisher’s estimate was merely a re-presentation. But numbers – even the *same* numbers – can mean different things. Punnett’s told of the long-term futility of a programme of sterilising ‘defectives’. The very same set of numbers, in Fisher’s hands, spoke to its immediate efficacy. Their openness to multiple interpretations and conflicting narratives means that even numbers designed to foreclose dissent can positively attract it. Numbers are powerful, but also vulnerable. The Eugenics Society came to realise this only too late.

Fisher’s number was a peculiar marriage of precision and vagueness. It made a highly specific prediction concerning not a concrete group about whom data had been systematically gathered, but rather an imagined, abstract population about which little was stipulated. Numbers such as these are powerful in their generality, while at the same time vain and potentially harmful in their detachment from the experiences and realities of relevant individuals. In the remainder of the article, we follow the Eugenics Society’s sterilisation campaign into the 1930s. This period saw a significant shift in the nature of their propaganda messaging, alongside the emergence of a dialogue between the eugenicists and the disabled populations which their proposed legislation targeted. Highlighting the agency of disabled people within the ongoing sterilisation campaign places in sharp focus the shortcomings of the eugenicists’ numerical rhetoric. It also necessitates that we rethink our understanding of the interactions between two groups, which seem at first to be antagonists, but which turn out to be complexly entangled.

### Disabling eugenics

Through the interwar period, some of the most important genetic work done on mental deficiency was instigated and informed by people who understood themselves as having personal stakes in these projects. For example, influential research on X-linked mental deficiency was initiated by a woman who was concerned about passing down such a condition to her children.^38^ She independently contacted Julia Bell (1879–1979), a researcher on the *Treasury of Human Inheritance* project, a long-term mass data-gathering exercise aimed at collecting and presenting material to illustrate human inheritance for students of heredity. It formed part of a much broader, connected body of research into inheritance in the first half of the twentieth century, administrated by multiple institutions, including the Galton Laboratory at University College London, the National Hospital, and the Medical Research Council. A major part of Bell’s work involved verifying the family pedigrees that had been created using data from individuals, doctors, specialists, academics, and various social and medical institutions across Britain. It was through her work with the National Hospital that Bell was put in touch with a mother who feared her son was ‘showing the signs of mental deficiency which she had already seen develop in some of the sons of her sisters.’^39^ This unknown mother worked extensively and collaboratively with Bell, her colleague James Purdon Martin, and her extended family to provide proof of a sex-linked mental deficiency. This is now known as fragile X syndrome, termed Martin-Bell syndrome when the paper was published in 1943, and this woman and her sisters were likely carriers.^40^

How should we think about this woman? Her case chimes with recent calls for a ‘disability turn’ in the history of science, wherein we ‘turn disability history to the topic of scientific knowledge production’.^41^Although anonymised as IV22, she is clearly more than an invisible assistant, having initiated the research project and provided all its data. It is clear, too, that the *Treasury of Human Inheritance* project required a certain amount of collaboration from the families who were the subjects of the pedigrees it created. Although analyses of large data sets have tended to focus more on digital tools and technologies, projects like the *Treasury* exemplify the need to focus on paper tools and their users.^42^ Their production and their use should be considered as collaborative – the writers were not the only figures that made this database work. Historians of science have long noted the inextricability of data and the scientists who produce it.^43^ But in the case of the human sciences, not only are the data and the scientist entangled – often, so too are their subjects.

Existing scholarship has sought to understand British eugenics through a range of lenses, including those of race,^44^ class,^45^ and gender.^46^ While each of these perspectives provides crucial insight, it is increasingly clear that *disability* occupied a central, if somewhat underappreciated, space in the eugenic project. Recently, the disability historian Michael Rembis asked what a history of eugenics might look like if scholars challenged traditional approaches, placing ‘disabled subjects of eugenic discourse at the center of the analysis’.^47^ We seek to answer Rembis’s challenge by focusing attention on disabled individuals who engaged with and responded to the Eugenics Society’s well-publicised campaign to legalise eugenic sterilisation during the 1930s. For some of these individuals, sterilisation represented a possible means by which to take charge of their own bodies and reproductive lives. Not merely passive targets of the Eugenics Society’s sterilisation discourse, disabled people were active participants and, as we will see, occasionally key contributors.

Centring *people* provides a stark contrast with what we might call, to appropriate Ted Porter’s phrase, the ‘quantitative impersonality’ of the eugenicists’ numerical rhetoric, with its strong tendency to divorce numbers from those individuals about whom they were made.^48^ We have already seen how interwar psychiatric practitioners drew on their daily interactions with ‘defective’ individuals, and resultant knowledge of their lives, to challenge the veracity of the eugenicists’ numbers. Letters sent to the Eugenics Society amid its sterilisation campaign provide an opportunity to focus attention on a group that has been denied a voice by both historical and historiographical forces, and in turn to deconstruct the simplistic categories upon which the Society’s campaign, and its calculations, were founded.^49^

### Eugenics by letters

At some point during the course of her involvement in Bell and Martin’s research, the woman anonymised as IV22 requested and received a sterilisation operation. We can only speculate as to the reasons for her decision. Perhaps she was encouraged to do so by her scientist-collaborators, or perhaps her thinking was influenced by the pro-sterilisation eugenic rhetoric, seventeen per cent and all, which was so visible through the interwar years. She was just one of many people who, throughout the 1930s, attempted to secure their own sterilisation. Many of these individuals, having encountered their propaganda messaging in the national press and elsewhere, reached out to the Eugenics Society for assistance, penning letters which are vulnerable and often heart-breaking, comparable in their candidness to those written to Marie Stopes seeking information and guidance on birth control methods.^50^ This correspondence, collected together in several folders within the archive of the Eugenics Society, reveals individuals who sought sterilisations for varied reasons, only sometimes aligning with the Society’s stated intentions for its use. As far as we are aware, these letters have not previously been subject to extended historical analysis, even while the history of the Eugenics Society – and of its sterilisation campaign – has been told and retold; an absence which exemplifies the wider neglect of disability in much past eugenics historiography.

In responding to approaches from members of the public – as it unfailingly did – the Eugenics Society always emphasised the present legislative difficulties, pointing out that only therapeutic sterilisation, when the procedure promised to improve the health of an individual, was definitively authorised. Most surgeons were therefore unwilling to risk the legal backlash which might arise from performing sterilisations in public hospitals, those being accountable to local authorities and, ultimately, taxpayers. Nevertheless, some were known to be willing to administer the operation privately for a fee, and in certain cases, the Society put correspondents in touch with such practitioners. When the individual concerned was poor and exhibited a ‘defect’ which was demonstrably hereditary, the Society would sometimes actively oversee the arrangement of the procedure, and occasionally cover the surgeon’s fee. For instance, the Society saw fit to intervene in the case of a Mrs B who had fallen pregnant, having previously birthed two sons with muscular dystrophy, and who threatened suicide if forced to carry the pregnancy to term. Following a referral from her doctor, C. P. Blacker, General Secretary of the Eugenics Society, lined up a London surgeon who agreed to abort the pregnancy, then sterilise the woman.^51^

Other similarly desperate women were judged not to meet the exacting criteria. One correspondent, Mrs S, wrote to Blacker on July 12 1931, explaining that

there has been mental trouble in my mother’s family I believe for some generations, two aunties having died in the asylum. My sister’s daughter, age 10 years is unable to read or write, and is getting more feeble minded as time goes on. As I and my husband do not wish to have a family, under the circumstances, we have been living apart for 8 years.^52^

She went on to ask for help in procuring a sterilisation operation that would allow her to reunite with her husband. In principle, the Society was enthusiastic about extending sterilisation to ‘normal people who might pass on some serious defect which is in their family’.^53^ However, the prevailing legal situation rendered such cases ineligible, and in responding to the woman, Blacker emphasised that her family history was not enough unless ‘you yourself suffered from some disability or ailment’.^54^ Four years later, another woman with a history of cleft lips in her and her husband’s family wrote that, ‘When my baby was born – 18 months ago – he was exactly like my husband’s uncle. Although we had a specialist’s advice and a wonderful operation was performed, he died at the age of 3 months’.^55^ The writer laments that her doctor had told her, ‘this deformity is inherited according to the laws of Mendel and that there is a very big danger of any other children I may have being like the first.’ She and her husband were united, she emphasised, in their decision ‘not to bring children into the world unless they stand a reasonable chance of being physically perfect’.^56^ In the two letters she wrote to the Society she uses the terms ‘carrier’ and ‘inherited’ and alludes to Mendel, but also offers an alternative explanation for the trait carried through the family; that her husband’s Grandmother was forced to skin a rabbit and that her own Mother saw a child fall and cut his lip early on in pregnancy. These stories, passed down through the generations, offered an alternative and potentially more optimistic diagnosis, and co-existed alongside the more fatalistic understanding of heredity that the Society transmitted through its propaganda. Evidence of established narratives circulating within families demonstrates both medical pluralism and the kinds of ‘folk medicine’ practised by ‘non-patients’ that Michael Worboys has recently suggested we should take more seriously.^57^ Some individuals evidently saw in sterilisation an escape from generational imperfection, whether they believed the cause to be genetic or otherwise.

Men, too, frequently wrote in asking for help to be sterilised, and for varied reasons. One explained that, ‘[A]lthough there is a bad history of consumption in my family, I foolishly got married, thinking I would be strong willed enough to avoid having any family myself’.^58^ Another sought sterilisation because he suffered from wet dreams, a condition he believed had been passed on to him through the ‘unclean living’ of his Father. And yet another man who wrote to enquire about sterilisation felt that he ‘ought to add that my wife and I and all our children are A1 lives.’^59^ Not all of those who reached out to the Society, then, exhibited hereditary defects, and this despite pro-sterilisation propaganda to date having focused so squarely upon the problem of inherited mental deficiency. In some cases, correspondents were suffering from infectious diseases such as syphilis.^60^ In others, a wife’s fragile health prompted her or her husband to seek sterilisation to ensure against future pregnancies.^61^ In still others, absent any health concerns hereditary or otherwise, sterilisation was pursued as a more reliable alternative to birth control, access to which was still patchy.^62^ Though the Eugenics Society was clear in its conviction that sterilisation should not be regarded as a substitute for birth control, many among the general public clearly disagreed. The letters taken together provide a snapshot of the diverse reasons that individuals sought sterilisations. While some expressed eugenics-inflected concerns about passing on hereditary afflictions, many others did not. As historian Jane Carey has observed for this period, the complex entanglements of reproductive decision-making with issues of race, class, and gender ensure that ‘the line between birth control and eugenics is actually impossible to draw’.^63^ This was particularly notable in the case of a young man of thirty who wrote in to explain that he was ‘the result of a mixed marriage. Besides being a little dark, I suffer with weak eyes compelling me to wear glasses for life’. As he had also developed osteomyelitis, he accordingly requested sterilisation to avoid bringing children into the world to face these health complaints alongside ‘racial troubles.’^64^ This letter is a striking example of the intertwining of race and disability, and the ways in which race was considered disabling in and of itself in the context of eugenic thinking.^65^

Of particular interest for our purposes are such exchanges with historical actors who considered *themselves* to be disabled and wished to structure their families accordingly. Letters held within the Eugenics Society archive record efforts by such individuals to claim reproductive agency, and point towards a complex, uneasy, and perhaps unexpected relationship between disabled people and the organised eugenics movement. It was, moreover, these individuals whom the Eugenics Society was most keen to help, and whose stories of struggles to access sterilisation would in turn prove useful. Indeed, through the 1930s, the Society would experiment with a novel form of eugenic propaganda, centred on qualitative and narrative accounts of affected individuals, as contrasted with the impersonal and dehumanising numerical arguments which dominated its earlier outputs. The letters privately sent to the Society are collected in several folders spanning the 1930s and 1940s. Within the files examined, there are forty-seven enquiries about sterilisation between 1930 and 1936.^66^ Of these approaches, nine came from people who explicitly considered themselves to be disabled in some way and sought sterilisations specifically to avoid passing on an undesired heritable condition.

These batches of carefully catalogued correspondence capture the patchwork efforts to enact ‘eugenic’ sterilisations in the absence of enabling legislation. From the Eugenics Society’s perspective, the letters also represented a repository of cases – an epistolary database – which it might, and did, mine for propaganda purposes in the course of its ongoing campaign. This function is exemplified by the case of Mr H, whose voluminous correspondence with the Society occupies a sub-folder of its own. Mr H first contacted the Eugenics Society in late 1930 after reading ‘Prof. Julian Huxley’s enlightening article’ published in *The Daily Mail*.^67^ He requested a copy of the Committee for Legalizing Voluntary Sterilization’s pamphlet mentioned in Huxley’s article, and went on to explain that he was ‘one of the victims awaiting legislation on this vital subject,’ owing to a ‘congenital deformity of both hands and feet’. Despite medical assurances that his condition would not be transmitted, the latest of his six children – the first five having been ‘perfectly formed’ – was ‘deformed in almost the same manner as myself’. Mr H and his wife were ‘anxious’, and he was ‘willing to undergo any operation in order that there shall never be any danger of another child coming into the world handicapped’.^68^

Blacker’s reply was enthusiastic. He sent H various pamphlets, and set about lining up a surgeon ‘who might see his way to sterilizing you’.^69^ Doing so was not straightforward, but as we shall see, this ultimately played to Blacker’s advantage. His first approach was to a surgeon at London’s Guy’s Hospital, who refused on the grounds of the questionable legality of performing such a procedure in a public hospital.^70^ Undeterred, Blacker eventually secured an appointment with a Leicester-based practitioner willing to conduct the sterilisation privately. Initially, H’s limited means left him unable to take up the offer, until several Eugenics Society Council members dipped their hands into their own pockets to cover the costs of the hospital stay and the surgeon’s fee. The operation took place on February 3 1931, with, as Blacker reported to readers of *The Eugenics Review*, ‘very satisfactory results’.^71^

Why did Blacker and colleagues go to so much effort and incur such personal financial expense to assist this man? If the guiding aim of sterilisation was to bring about ‘racial progress’, then the prevention of one or two hypothetical future children with possible physical defects was almost inconsequential. Doubtless, there was personal satisfaction in helping couples negotiate sometimes desperate situations. Nevertheless, the Society’s efforts, for which the Hs expressed extreme gratefulness, were calculated. From the outset, Blacker identified H’s case as such a good one that it might usefully be made an example of. His efforts in this direction were aided by Mr H’s willing cooperation throughout, motivated, it seems, by a genuine interest in and commitment to eugenic principles. While still scrambling to secure a willing surgeon, Blacker requested permission to give ‘publicity’ to the letters, which Mr H gladly granted, explaining: ‘Quite apart from my own personal need I have always taken a keen interest and am entirely in favour of the proposals of your Society’.^72^ At first, this ‘publicity’ was largely internal, as Blacker summarised H’s case and excerpted his correspondence in the *Eugenics Review*.^73^ The coming months would see his ambitions and his audience expand considerably.

1931 was also the year that the Eugenics Society eventually succeeded in convincing a sitting member to bring their Sterilisation Bill to parliament; on July 21, it was introduced under the ten-minute rule by Major A. G. Church, member for Wandsworth Central.^74^ The Bill proposed that the surgical sterilisation of certified ‘mental defectives’ be made legal on a ‘voluntary’ basis, requiring consent from the patient and their family, as well as the approval of medical representatives of the Board of Control for Lunacy and Mental Deficiency. It was defeated comfortably, eighty-nine votes in favour versus 167 against, with 130 of the opposers coming from the Labour Party. Among the stated reasons for opposition was the doubt that individuals deemed ‘mentally deficient’ could knowingly consent to their own sterilisation. One way that eugenicists hoped to deflate this principled objection was to move focus away from intellectual disabilities and towards cases of mentally ‘normal’ individuals who desired, but were effectively debarred from accessing, sterilisation. And so, several months after their last contact, Blacker reached out again to Mr H, in the hope that his testimony could help shift the public and political conversation. It required some creative framing, with Blacker explaining to his correspondent that, although the defeated Bill had concerned only the ‘mentally deficient’, it had always been intended as the ‘thin end of a wedge, the thick end of which would be a Bill with wider scope to legalise the sterilization of persons afflicted with hereditary diseases and defects such as you yourself suffer from’.^75^ Indeed, that Mr H was of sound mind (and wrote eloquently about his situation) was strategically important. This meant, for example, that any concerns about consent or coercion could be safely bypassed. Here was a man who seemed to genuinely want to be sterilised, for what many people at the time would have agreed were good reasons. Thus, ironically, the fact that he *was not* intellectually disabled proved useful in the Eugenics Society’s attempts to gain support for a law which they had initially supported for reasons of sterilising specifically *intellectually* disabled people.

Besides the vexed issue of consent, the other major sticking point was the Bill’s class politics. Certain detractors, Blacker noted, had attempted to paint the Bill as ‘anti-working class legislation’, and their success in doing so was borne out by the preponderance of Labour MPs among those who opposed it. Mr H, as a working man, could again provide a useful counter, and help the Society shake off its (well-earned) reputation for classism and dispel the notion that eugenics was necessarily oppositional to the interests of the poor. To ‘victimize the poor’, Blacker assured him, was not the aim. On the contrary, the Eugenics Society merely desired to ‘confer upon them a benefit now almost exclusively enjoyed by the rich, who can afford to pay surgeon’s fees’.^76^ The determination of the Eugenics Society through the 1930s to reach out to the organised Labour movement, and to sow approval for voluntary sterilisation among the working classes, has attracted attention from historians including Greta Jones and, more recently, David Redvaldsen.^77^ These studies provide a vivid portrait of the Society’s concerted conciliatory mission, consisting of tireless lecture circuits of provincial working women’s groups and trade union branches. However, they overlook the vital role played by the Society’s tactical deployment of personal testimonies by its disabled correspondents.^78^

The ‘preliminary measure’ which Blacker put before Mr H was for the latter to send a letter into the left-leaning mass-circulation newspaper *The Daily Herald*, detailing his struggles as a poor man in accessing the sterilisation procedure – so easily available to those better-off – which would ensure that his hereditary disablements were not transmitted further. Blacker stage-managed the whole affair. He drafted the letter, which explained how the Eugenics Society had helped H by paying his sterilisation fee, to his great relief and to the end of making ‘available for the poor what is now the privilege of the rich’. Blacker also instructed his correspondent when and where to send the letter, and even suggested possible pseudonyms should the ‘author’ wish to retain his anonymity. (Blacker offered ‘Hereditarily afflicted’; H eventually settled on ‘Hereditary deformity’).^79^ In the event, both *The Daily Herald* and second choice *The Daily Mail* passed on publication, and months would pass before the letter was placed successfully in *The Week-End Review*, appearing on 14 May 1932. While they hoped the letter would make a splash (hence the decision to delay until after the autumn general election, when press and public attention would be otherwise occupied), any conversation it sparked in the newspapers would be a bonus. It was always Blacker’s primary intention that H’s letter serve as a more permanent piece of propaganda. So long as it *was* published, anywhere, the Eugenics Society could reprint the letter as a leaflet for distribution in great numbers at its lectures and meetings. This is just what they did throughout the 1930s.^80^

‘The best eugenic propaganda at the present moment is a bare statement of ascertained facts’.^81^ So claimed a memorandum, penned in early 1930 as the Eugenics Society was still considering the best course of response to the 1929 Wood Report on mental deficiency, and signed by both Fisher and Blacker. Over the next months and years, the Society’s propaganda activities would demonstrate just how far this seemingly straightforward sentiment masks a host of complexities. As we have seen, ‘facts’ could and did mean many things, from contested statistics to choreographed personal testimonies. The routes through which they were ‘ascertained’ were anything but simple. It should be clear, also, that their use rarely amounted to ‘bare statement’. Finally, which sorts of facts might turn out to be the ‘best’ propaganda was hotly debated and proved difficult to predict.

Placing Mr H’s letter and Fisher’s 17.4 per cent figure side-by-side is illuminating. Both are instances of eugenic propaganda, which elevated a single datum which the Eugenics Society deemed especially salient and sought to communicate widely through pamphlets and other printed outputs. At the same time, the two cases differ markedly in style, substance, audience, and purpose. H’s letter is an attempt to claim the authority, not of the statistical expert, but rather of the sufferer of hereditary disease, the ordinary person struggling to lay claim to their reproductive agency. It was intended to win over not the medical establishment, which had opposed the Eugenics Society’s sterilisation proposals on scientific and clinical grounds, but rather the Labour movement, which had resisted them as a matter of class politics. The campaign to legalise eugenic sterilisation underwent a significant shift across the period treated in this article. In the early years, up to around 1931, sterilisation was conceived and presented by eugenicists as a tool for battling a growing epidemic of hereditary disease, and for the scientific management of a population through the ‘elimination of mental defect’. The Eugenics Society, in its public pronouncements, leant heavily on numbers and statistics which were divorced from the complex realities of people living with these conditions. Through the rest of the 1930s, the Society attempted to reposition their campaign as a crusade to rectify a legal situation which ‘discriminates against poor people’ who, for structural and financial reasons, were effectively barred from accessing a procedure which would help them achieve reproductive autonomy.^82^ At the same time, they explicitly widened the scope of who should be sterilised – from ‘mental defectives’ to people living with a much broader range of hereditary ‘defects’. As they did so, abstract numbers faded from view, while disabled individuals and their stories came to the fore.

### Epilogue: data afterlives

In July of 2020, at the height of global Black Lives Matter protests, the Council of Gonville and Caius College, a constituent college of the University of Cambridge, resolved to remove from the College’s dining hall a stained-glass window commemorating the remarkable scientific contributions of former student and Fellow, Ronald Aylmer Fisher. The Council’s decision was prompted by a student-led petition, which determined to shine a light on Fisher’s racism, as well as his lifelong eugenic advocacy.

As the petition circulated online and accrued digital signatures, the celebrated historian of Nazi Germany Sir Richard Evans – a Fellow of the College sympathetic to the students’ campaign – penned a supportive essay, published online by the *New Statesman*. The piece detailed many of the more unsavoury aspects of Fisher’s activities and views, including his close involvement in the sterilisation campaign, which was spotlighted in the article’s tagline. At one point, Evans writes:

The extent to which the *17 per cent of the British population estimated by Fisher to be* ‘*defectives*’ were capable of objecting to their own ‘voluntary’ sterilisation when advised to do so by medical authorities must be extremely doubtful.^83^

In the course of legitimately questioning the sincerity of the eugenicists’ rhetorical insistence on the *voluntary* nature of their proposed measures, Evans fundamentally mischaracterises the nature of Fisher’s number. In no way an estimate of prevalence, it was rather a projection, quantifying the expected reduction of incidence upon sterilisation of ‘defectives’. This would hardly be remarkable if it were a one-off. Yet, Evans’s is merely the latest iteration of a long-standing pattern of misinterpretation within the historical literature on interwar eugenics. In his 1989 article on the sterilisation campaign in interwar Britain, historian John Macnicol has seventeen per cent as Fisher’s personal estimate of ‘the proportion of mental defectives who owed their condition to heredity’.^84^ King and Hansen go one better in their 1999 study. Using Macnicol as their source, they give the number the same erroneous meaning, while misascribing it to its greatest detractor, A. F. Tredgold.^85^ As we have seen, the brief heyday, circa 1930, of Fisher’s 17.4 per cent estimate was characterised by ambiguity and uncertainty about precisely what this number meant. This confusion about the nature and proper interpretation of the statistic never went away. Neither did the number itself. It continued to circulate in new contexts, its meaning constantly remade. As Ted Porter has recently put it, when it comes to the journeys of data, numbers, and statistics, ‘[m]ost often, what is transmitted is transformed’.^86^

Though disposed of as quickly as it was taken up by sterilisation campaigners, Fisher’s figure has enjoyed – or rather, endured – a long and curious afterlife. Despite its dismissal, first by critics of sterilisation and soon after by advocates, the number refuses to go away. Its various misreadings become part of the number’s long history – its flexibility and ambiguity at first underlay its power, and later precipitated its downfall. Now, they fuel its persistence. Contrast this with the letters from individuals who, through the 1930s, reached out to the Eugenics Society seeking sterilisations. These letters, even those like Mr H’s which the Eugenics Society shamelessly choreographed and reproduced as propaganda material, are long forgotten. It is striking that impersonal numbers such as Fisher’s have been given new lives (and new meanings) in generations of scholarship and writing on the sterilisation movement, while Mr H’s letter, despite serving an equally significant propaganda role, has remained buried – preserved by archivists, custodians of data, but unremarked upon by historians.

## Footnotes

1 Francis Galton, *Probability, the Foundation of Eugenics: The Herbert Spencer Lecture delivered on June 5, 1907* (Oxford: The Clarendon Press, 1907), 13.

2 For a recent survey of the history of eugenics in Britain, see: David Redvaldsen, *A History of British Eugenics since 1865: From Francis Galton to Designer Babies* (Cham: Palgrave Macmillan, 2024). For the pre-Galtonian ‘pre-history’ of eugenics in Britain, see: John C. Waller, ‘Ideas of heredity, reproduction and eugenics in Britain, 1800–1875’, *Studies in History and Philosophy of Science Part C: Studies in History and Philosophy of Biological and Biomedical Sciences*, 32, 3 (2001), 457–89.

3 For a useful collection of essays on the diversity of eugenics movements across varied locales, see: Alison Bashford and Philippa Levine (eds), *The Oxford Handbook of the History of Eugenics* (Oxford: Oxford University Press, 2010). For a perspective which approaches eugenics through its manifestation in several British colonies, see: Diane B. Paul, John Stenhouse and Hamish G. Spencer (eds), *Eugenics at the Edges of Empire: New Zealand, Australia, Canada and South Africa* (Cham: Springer, 2018).

4 On the Eugenics Society (founded in 1907 as the Eugenics Education Society, and renamed in 1926), see: Pauline M. H. Mazumdar, *Eugenics, Human Genetics and Human Failings: The Eugenics Society, its Sources and its Critics in Britain* (London: Routledge, 1991). On the Society’s failed campaign for the introduction of eugenic family allowances, see: Alex Aylward, ‘R. A. Fisher, Eugenics, and the Campaign for Family Allowances in Interwar Britain’, *The British Journal for the History of Science*, 54, 4 (2021), 485–505.

5 Though see, e.g., Erika Dyck, *Facing Eugenics: Reproduction, Sterilization, and the Politics of Choice* (Toronto: University of Toronto Press, 2013); Molly Ladd-Taylor, ‘Contraception or Eugenics? Sterilization and “Mental Retardation” in the 1970s and 1980s’, *Canadian Bulletin of Medical History*, 31, 1 (2014), 189–211.

6 Coreen McGuire, *Measuring Difference, Numbering Normal: Setting the Standards for Disability in the Interwar Period* (Manchester: Manchester University Press, 2020).

7 For early and influential statements of this thesis, see: Ruth Schwartz Cown, ‘Francis Galton’s Statistical Ideas: The Influence of Eugenics’, *Isis*, 63, 4 (1972), 509–28; Donald MacKenzie, *Statistics in Britain, 1865*–*1930: The Social Construction of Scientific Knowledge* (Edinburgh: Edinburgh University Press, 1981). See also: Francisco Louçã, ‘Emancipation through Interaction – How Eugenics and Statistics Converged and Diverged’, *Journal of the History of Biology*, 42, 4 (2009), 649–84.

8 On anthropometry in the service of eugenics, see: Elise Smith, ‘Class, Health and the Proposed British Anthropometric Survey of 1904’, *Social History of Medicine*, 28, 2 (2015), 308–29; ‘“Why do we measure mankind?” Marketing Anthropometry in late-Victorian Britain’, *History of Science*, 58, 2 (2020), 142–65. On eugenicists’ deployment of demographic statistics, see: Richard A. Soloway, ‘Counting the Degenerates: The Statistics of Race Deterioration in Edwardian England’, *Journal of Contemporary History*, 17, 1 (1982), 137–64; *Demography and Degeneration: Eugenics and the Declining Birthrate in Twentieth-Century Britain* (Chapel Hill: University of North Carolina Press, 1990); Vanessa Heggie, ‘Lies, Damn Lies, and Manchester’s Recruiting Statistics: Degeneration as an “Urban Legend” in Victorian and Edwardian Britain’, *Journal of the History of Medicine and Allied Sciences*, 63, 2 (2008), 178–216.

9 Graham J. Baker, ‘Eugenics and Migration: A Case Study of Salvation Army Literature about Canada and Britain, c. 1890–1921’, *Canadian Bulletin of Medical History*, 31, 1 (2014), 77–98.

10 David Barker, ‘The Biology of Stupidity: Genetics, Eugenics and Mental Deficiency in the Inter-War Years’, *The British Journal for the History of Science*, 22, 3 (1989), 347–75.

11 Mathew Thomson, ‘Sterilization, Segregation and Community Care: Ideology and Solutions to the Problem of Mental Deficiency in Inter-War Britain’, *History of Psychiatry*, 3 (1992), 473–98; *The Problem of Mental Deficiency: Eugenics, Democracy, and Social Policy in Britain, c. 1870-1959* (Oxford: The Clarendon Press, 1998).

12 Bradley W. Hart and Richard Carr, ‘Sterilization and the British Conservative party: Rethinking the Failure of the Eugenics Society’s Political Strategy in the Nineteen-Thirties’, *Historical Research*, 88, 242 (2015), 716–39.

13 William Alonso and Paul Starr (eds), *The Politics of Numbers* (New York: Russell Sage Foundation, 1987); Andrea Mennicken and Robert Salais (eds), *The New Politics of Numbers: Utopia, Evidence and Democracy* (Cham: Palgrave Macmillan, 2022).

14 The single extended study of the rhetoric of anglophone eugenics, for example, says almost nothing about *numerical* rhetoric – see: Marouf Anif Hasian, *The Rhetoric of Eugenics in Anglo-American Thought* (Athens: University of Georgia Press, 1996).

15 Other scholars have begun, in similar ways, to follow the rise, circulation, and fall of individual numbers. See e.g., Andrea Bréard (ed), ‘Focus: Global Life Histories of Numbers’, *Isis*, 116, 1 (2025), 123–67.

16 Theodore M. Porter, *Trust in Numbers: The Pursuit of Objectivity in Science and Public Life* (Princeton: Princeton University Press, 1995).

17 Frank A. Gill, ‘Sterilisation of the Unfit: A Report to the Lancashire Asylums Board’, *The Lancet*, 21 June 1930, 1380–2 (p. 1381).

18 For a recent history of the Mental Deficiency Act, see Sarah Wise, *The Undesirables: The Law that Locked Away a Generation* (London: Oneworld, 2024). On the Eugenics Society’s (limited) role in the passage of the act, see: Edward J. Larson, ‘The Rhetoric of Eugenics: Expert Authority and the Mental Deficiency Bill’, *The British Journal for the History of Science*, 24, 1 (1991), 45–60.

19 Paul A. Lombardo, *Three Generations, No Imbeciles: Eugenics, the Supreme Court, and Buck v*. Bell (Baltimore: Johns Hopkins University Press, 2022).

20 According to the Wood Report, the numbers of certified ‘defectives’ rose from 4.6 per thousand in 1909 to 8.56 per thousand in 1929; *Report of the Mental Deficiency Committee, Being a Joint Committee of the Board of Education and Board of Control* (London, 1929).

21 Mathew Thomson has noted that ‘[u]ndoubtedly, the unwillingness of the British Medical Association to back the campaign [for sterilisation] was damaging’, *The Problem of Mental Deficiency*, 195.

22 Bernard Mallett to Ronald Aylmer Fisher, 8 July 1930, Eugenics Society Papers, Wellcome Library (hereafter ESP), SA/EUG/C.107.

23 Gill, ‘Sterilisation of the Unfit’, 1381.

24 ’Sterilisation of the Unfit’, *The Lancet*, 19 July 1930, 161. The letter to the *BMJ* contained an almost identically worded statement – see: ‘Sterilization of the Unfit’, *The British Medical Journal*, 26 July 1930, 159–60 (p. 160).

25 Barker, ‘Biology of Stupidity’.

26 Reginald C. Punnett, ‘ELIMINATING FEEBLEMINDEDNESS: Ten Per Cent of American Population Probably Carriers of Mental Defect—If Only Those Who Are Actually Feebleminded Are Dealt with, It Will Require More Than 8,000 Years to Eliminate the Defect—New Method of Procedure Needed’, *Journal of Heredity*, 8, 10 (1917), 464–5.

27 Diane B. Paul and Hamish G. Spencer, ‘Did Eugenics Rest on an Elementary Mistake?’, in Rama S. Singh, et al. (eds), *Thinking About Evolution: Historical, Philosophical, and Political Perspectives* (Cambridge: Cambridge University Press, 2001), 103–18 (pp. 108–9).

28 R. A. Fisher, ‘The Elimination of Mental Defect’, *The Eugenics Review*, 16, 2 (1924), 114–6.

29 Cora Hodson [Eugenics Society Secretary] to R. A. Fisher, 8 August 1927 in ESP, SA/EUG/C.107.

30 Mallet to Fisher, 8 July 1930, ESP, SA/EUG/C.107.

31 *Committee for Legalising Eugenic Sterilization* (London: The Eugenics Society, 1930). A copy of this pamphlet is held in ESP, SA/EUG/J.18.

32 Mallet to Fisher, 8 July 1930, ESP, SA/EUG/C.107.

33 Fisher to Mallet, 13 October 1930, ESP, SA/EUG/C.107.

34 ’Eugenic Sterilisation’, *The Lancet*, 16 August 1930, 369–70; ‘The Legalisation of Eugenic Sterilisation’, *The Lancet*, 16 August 1930, 360.

35 Typescript of C. P. Blacker speech delivered at the CAMW Conference on Mental Welfare, 11–13 December 1930. Copy in ESP, SA/EUG/D.53. See: ‘Conference on Mental Welfare’, *The British Medical Journal*, 2, 3650 (1930), 1058–60.

36 C. P. Blacker to Julian Huxley, 24 October 1930, ESP, SA/EUG/C.185.

37 Mallet to Fisher, 26 November 1930, ESP, SA/EUG/C.107.

38 J. Purdon Martin and Julia Bell, ‘A Pedigree of Mental Defect Showing Sex-Linkage’, *Journal of Neurology and Psychiatry*, 6, 3–4 (1943), 154–7.

39 Martin and Bell, ‘A Pedigree of Mental Defect’, 154.

40 Martin and Bell, ‘A Pedigree of Mental Defect’; Jesse King, ‘Julia Bell (1879–1979)’, *Embryo Project Encyclopaedia* (2012-12-27). ISSN: 1940-5030 http://embryo.asu.edu/handle/10776/4209.

41 Mara Mills, Jaipreet Virdi, and Sarah F. Rose, ‘Disability, Epistemology, Sciencing’, *Osiris*, 39, 1 (2024), 2–24 (p. 24).

42 C. Bittel, E. Leon & C. von Oertzen (eds), *Working and Knowing with Paper: Gendered Practices in the History of Knowledge* (Pittsburgh: University of Pittsburgh Press, 2019), 1. For a study of digital datasets in the context of heredity, see: Michael F. McGovern, ‘Genes Go Digital: *Mendelian Inheritance in Man* and the Genealogy of Electronic Publishing in Biomedicine’, *The British Journal for the History of Science*, 54, 2 (2021), 213–31.

43 Lorraine Daston and Peter Galison, *Objectivity* (New York: Zone Books, 2007), 232.

44 Dan Stone, ‘Race in British Eugenics’, *European History Quarterly*, 31, 3 (2001), 397–425; *Breeding Superman: Nietzsche, Race and Eugenics in Edwardian and Interwar Britain* (Liverpool: Liverpool University Press, 2002).

45 Donald MacKenzie, ‘Eugenics in Britain’, *Social Studies of Science*, 6, 3–4 (1976), 499–532.

46 Angelique Richardson, *Love and Eugenics in the Late Nineteenth Century: Rational Reproduction and the New Woman* (Oxford: Oxford University Press, 2003).

47 Michael Rembis, ‘Disability and the History of Eugenics’, in Michael Rembis, Catherine Kudlick and Kim E. Nielsen (ed), *The Oxford Handbook of History of Disability* (Oxford: Oxford University Press, 2018), 85–104 (p. 85).

48 Theodore M. Porter, ‘Making Things Quantitative’, *Science in Context*, 7, 3 (1994), 389–407 (p. 393).

49 Michael Rembis, ‘(Re)Defining Disability in the ‘Genetic Age’: Behavioral Genetics, ‘New’ Eugenics and the Future of Impairment’, *Disability & Society*, 24, 5 (2009), 585–97.

50 See Claire Davey, ‘Birth Control in Britain during the Interwar Years: Evidence from the Stopes Correspondence’, *Journal of Family History*, 13, 1 (1988), 329–45.

51 Series of letters ending with a letter to Blacker dated Friday 26 April 1935, ESP, SA/ EUG/ D.210. Although most correspondents used their own names, we have taken the decision to anonymise them.

52 Letter from Mrs S to Dr Blacker, 12 July 1931, ESP, SA/ EUG/ D.210.

53 *Voluntary Sterilization* [pamphlet printed for the Joint Committee on Voluntary Sterilization], (London, 1934). Copy in ESP, SA/EUG/ D.229.

54 Letter from the General Secretary to Mrs S, 14 July 1931, ESP, SA/ EUG/ D.210.

55 Letter from Mrs E, 14 January 1935, ESP, SA/EUG/ D.211.

56 Letter from Mrs E, 14 January 1935, ESP, SA/EUG/ D.211.

57 Michael Worboys, ‘The Non-Patient’s View’, in A. Hanley and J. Meyer (eds), *Patient Voices in Britain, 1840*–*1948* (Manchester: Manchester University Press, 2021), 33–60.

58 Mr J to Blacker, 10 July 1933, in ESP, SA/EUG/D.210.

59 Letter dated June 5 (from response, 1936) indecipherable signature, ESP, SA/ EUG/ D.211.

60 Dr. Edwards to C. P. Blacker, 30 October 1931, ESP, SA/EUG/D.210.

61 For instance, one woman explained that she had recently been admitted to a ‘mental hospital with a complete nervous breakdown’ and her doctor had subsequently warned her not to ‘risk having another baby or I should break up again’; Letter from Mrs H, 25 January 1936, ESP, SA/ EUG/ D.211.

62 See, e.g., the exchange between Blacker and a Mr W, 12, 13 October 1931, ESP, SA/EUG/D.210.

63 Jane Carey, ‘The Racial Imperatives of Sex: Birth control and eugenics in Britain, the United States and Australia in the interwar years’, *Women*’*s History Review*, 21, 5 (2012), 733–52 (p. 736).

64 Letter from Mr S, 7 July 1935, ESP, SA/ EUG/ D. 211.

65 For a full discussion of this connection, see Dennis Tyler, *Disabilities of the Color Line: Redressing Antiblackness from Slavery to the Present* (New York: NYU Press, 2022).

66 Within the vastness of the Eugenics Society Papers there is a box of materials related to ‘voluntary sterilization’, which includes a folder titled ‘enquiries about operation’ containing several files between 1930 and 1948. These comprise the main source material for this section of the article. There are three relevant files with this title but the most recent file from 1937–1948 (SA/EUG/ D.212) is currently closed. As a result, we have focused on the letters contained within SA/ EUG/ D.210 1930–1934 and SA/ EUG/D, 211 1935–1936. We pay particular attention to letters in which the writer sought a sterilisation for themselves and considered themselves disabled. The files also contain many examples of people writing to try and procure sterilisations for varied reasons, for family members, patients, neighbours, and so forth.

67 The article in question is Julian Huxley, ‘One in 100 a Burden to the Rest’, *The Daily Mail*, 27 November 1930, 10.

68 Mr H to C. P. Blacker, [undated – late November 1930], ESP, SA/EUG/D.215.

69 Blacker to H, 2 December 1930, ESP, SA/EUG/D.215.

70 ’…I therefore feel, much as I agree with your views, that until your act is passed, as I hope it will be – one would not be justified in doing this operation in a public hospital’; E. G. Slesinger to Blacker, 9 December 1930, ESP, SA/EUG/D.215.

71 C. P. Blacker, ‘Sterilization of Poor Patients’, *The Eugenics Review*, 23, 1 (1931), 63–4 (p. 64).

72 Blacker to H, 1 January 1931 & H to Blacker, 2 January 1931, ESP, SA/EUG/D.215.

73 Blacker, ‘Sterilization of Poor Patients’.

74 Hart and Carr, ‘Sterilization and the British Conservative Party’, 723–4.

75 Blacker to H, 12 August 1931, ESP, SA/EUG/D.215.

76 Blacker to H, 12 August 1931, ESP, SA/EUG/D.215.

77 Greta Jones, *Social Hygiene in Twentieth Century Britain* (London: Croom Helm, 1986); David Redvaldsen, ‘The Eugenics Society’s Outreach to the Labour Movement in Britain, 1907–1945’, *Labour History Review*, 78, 3 (2013), 301–29; ‘Eugenics, Socialists and the Labour Movement in Britain, 1865–1940’, *Historical Research*, 90, 250 (2017), 764–87.

78 The Eugenics Society was not alone in adopting such tactics in interwar Britain. For instance, later in the decade the Abortion Law Reform Association would make significant use of personal testimonies detailing individual experiences of the abortion law – see: Susanne Maria Klausen, ‘Thorny Entanglements: Feminism, Eugenics and the Abortion Law Reform Association’s (ALRA) Campaign for Safe, Accessible Abortion in Britain, 1936–1967’, *Medical History*, 68, 1 (2024), 86–108.

79 Blacker to H, 12 August 1931, ESP, SA/EUG/D.215.

80 Letter signed by ‘Hereditary Deformity’, in folder containing ‘Miscellaneous items on sterilization’, ESP, SA/EUG/ D.229.

81 Memorandum presented to the Council in the form of a general recommendation by the Sterilisation Hospital Committee, in ‘Miscellaneous articles re Voluntary sterilisation 1930s–1950s’, in Carlos Blacker Papers, Wellcome Library, London, PP/CPB/ B.5/4. This Committee was set up in 1929, as the Eugenics Society (briefly) considered sponsoring the building of a specialised hospital for the sterilisation of ‘mental defectives’. Ambiguities over the legal standing of sterilisation – the same which would deter surgeons from performing the operation in public hospitals – soon put an end to the ambitious plan, and attention turned to lobbying for change in the law.

82 Blacker to H, 1 January 1931, ESP/EUG/D.215.

83 Richard Evans, ‘R. A. Fisher and the Science of Hatred’, *New Statesman*, published online 28 July 2020: https://www.newstatesman.com/long-reads/2020/07/ra-fisher-and-science-hatred. Accessed 20 July 2024. Emphasis added.

84 John Macnicol, ‘Eugenics and the Campaign for Voluntary Sterilization in Britain between the Wars’, *Social History of Medicine*, 2, 2 (1989), 147–69 (p. 159).

85 Desmond King and Randall Hansen, ‘Experts at Work: State Autonomy, Social Learning and Eugenic Sterilization in 1930s Britain’, *British Journal of Political Science*, 29, 1 (1999), 77–107 (p. 104). Fisher’s own best guess is given as five per cent.

86 Theodore M. Porter, ‘Most Often, What Is Transmitted Is Transformed’, in S. Leonelli and N. Tempini (eds), *Data Journeys in the Sciences* (London: Springer Nature, 2022), 229–36.

